# Dose Remittances Matter for Openness and Financial Stability: Evidence From Least Developed Economies

**DOI:** 10.3389/fpsyg.2021.696600

**Published:** 2021-08-10

**Authors:** Meng Miao, Md. Qamruzzaman

**Affiliations:** ^1^Renmin University of China, Hanqing Advanced Institute of Economics and Finance, Beijing, China; ^2^School of Business and Economics, United International University, Dhaka, Bangladesh

**Keywords:** remittances, economic openness, financial openness, financial stability, system-GMM

## Abstract

The study's motivation is to gauge the effects of remittances on openness: financial and economic openness and financial stability in least developed countries (LDCs) for the period spanning 1975–2018. The study applies Generalized Moment of Methods (GMM) and System-GMM to detect the magnitude of remittances, gross capital formation, and government debt on openness and financial stability, and their directional association is established by performing a Granger causality test with System-GMM specification. The results of cross-sectional dependency ascertain the presence of a common dynamic among the research units; on the other hand, both first, and second-generation unit root tests establish that variables are integrated either at level or after the first difference, neither variables are exposed to order of integration after second difference. A panel co-integration test based on error correction confirms the availability of the long-run association among variables. Study findings with GMM and System-GMM estimation expose positive statistically significant effects of remittance inflows to economic and financial openness and financial stability. In LDCs, remittance inflows positively augment economic and financial openness; moreover, financial stability remittances play a critical role. The study implemented the Granger causality test with System-GMM specification, and results disclosed the feedback hypothesis that is bidirectional causality availability in the tested empirical causal model.

**JEL Classifications:** F24, F43, P34.

## Introduction

Remittances to developing countries go first and foremost to lower-middle-income and low-income countries. Lower-middle-income countries receive the most considerable amounts. However, remittances constitute a much higher share of total international flows to low-income countries (Gammeltoft, 2002). The position of immigrant remittances in economic growth is still a hot topic among academics and policymakers. Also, all countries are interested in international aid remittances; since they represent a large influx of financial capital; both industrialized and emerging economies are drawn to each other (Chami et al., 2005; Stojanov et al., 2019). Furthermore, if made more explicit, immigrant remittances can even help grow nations or be channeled into profitable investment in the economy (Straubhaar, 1986; Elbadawi and Rocha, 1992; Chami et al., 2003; Ratha, 2005).

In recent times, remittances have been considered the critical source in foreign capital flows in the economy, especially in developing nations, about 27% of GDP. The steady growth of remittances helps accelerate domestic capital and allows a higher degree of technological advancement, which eventually reduces transaction costs (Bevan et al., 2004; Aggarwal et al., 2011; Sibindi and Bimha, 2014). Furthermore, Nyamongo et al. (2012) advocated that remittances are a significant source of savings and money for wellness, schooling, and development, resulting in increased production and jobs leading to economic development. Aggarwal et al. (2011) postulated that financial sector development through remittance could be observed through channelizing remittance into productive investment and capital accumulation.

In addition, remittances may represent capital flows that have yet to be determined, but the verdict is still out. It is because of the importance of government policy growth, and it is worth looking at. One way to do this is to see whether remittance behavior is similar to other forms of capital flows. Expressly, we assume that remittance flows would be positive with sustainable development through economic expansion (Qamruzzaman and Jianguo, 2020b) and financial diversification (Gnangnon, 2020; Pandikasala et al., 2021). Existing literature advocates that remittances are not sufficient for economic growth but rather work in a better manner in those economies with a higher degree of openness, indicating that benefit depends on domestic institutions and the receiving country's macroeconomic environment. Kapur and Singer (2006) showed that remittance inflows appear to minimize government consumption in developed countries and validate the replacement impact between private insurance given by remittances and public insurance initially provided by government expenditure. Furthermore, remittances mainly aim to take advantage of high yields or other home country investment prospects (Kumar, 2011). Even by lower barriers to trade, greater economic access could promote an increase in remittances that fund purchases of products and services required to sustain migrant associations' projects in the home countries (Kumar, 2013; Nahar et al., 2018).

The contribution from this study to the existing literature is three-fold. **First**, the impact of remittances on the economy, precisely focusing on both macro aspects, including economic, financial development (Ahmed et al., 2021; Bolarinwa and Akinbobola, 2021; Ellahi and Omer, 2021), financial inclusion (Issabayev et al., 2020; Barnabe, 2021), and macro aspects, such as household consumption (Raihan et al., 2009, 2021; Kumar et al., 2021), education (Zhunio et al., 2012; Ambler et al., 2015), and social security (De Haas, 2010; Peth and Sakdapolrak, 2020) have been investigated but there is yet any conclusive evidence. However, the impact of remittances is undoubtedly acknowledged and appreciated in literature from every corner of the economy. With this study, we intended to explore fresh evidence regarding the impact of remittances on economic openness and the role of establishing financial stability by considering a panel of 40 least developed countries (LDCs) for the period spanning 1975–2018. Second, the role of remittances is critically important for both developed and developing nations, but to what extent can remittances contribute to LDCs' development? No such focused research has yet been performed in empirical estimation. The existing research gap focuses on remittances' influence on unexposed LDCs; thus, we tried to lessen that gap with this study's fresh assessment.

The study's motivation is to evaluate the impact of remittances on openness, i.e., economic and financial openness and financial stability in LDCs, with the mediating effect of capital formation and government debt from 1975 to 2018. Study findings expose positive and statistically significant effects running from remittances to openness and financial stability. Findings suggest that continual remittance inflows assist in achieving openness in the economy through economic openness and financial openness. Furthermore, in establishing financial stability the role of remittances is critically essential. The remaining structure of the manuscript is as follows. The relevant literature survey is displayed in Section Literature review: remittance, openness, and financial stability, variable definitions and the econometrical methodology are explained in Section Data and methodology of the study. Empirical model estimation and interpretation are displayed in Section Estimation and interpretation, and finally, a summary of findings and the conclusion are in Section Findings and Conclusions.

## Literature Review: Remittance, Openness, and Financial Stability

The theoretical literature on remittances is extensive because several scholars have proposed hypotheses explaining their position in the economy, at least informally, to inspire an empirical study. However, several of the ideas presented have a similar thread running through them. Early theories of remittances established and explained a variety of costs and advantages associated with remitting. Russell (1986) compiled a list of them. Since the whole family shares and sells off the costs and advantages of remitting, the family is the optimal study for migration and remittance issues. As a result, recent theoretical work on remittances' nature has centered on and may be classified by the potential functions of family or family interactions in influencing remittance choices. There is still controversy about the idea of enabling foreign labor mobility as a way to boost remittance inflows to developed countries (Yang and Choi, 2007; De Haas, 2010; Bellantuono et al., 2021). Developing countries fret about a “brain drain,” even as remittances and commerce and investment are more than compensating for professional staff shortages abroad. A large increase in foreign migration may have significant economic advantages (Schiff and Özden, 2005). However, the adverse association between remittances and economic progress is also established in the literature; see, for instance, (Chami et al., 2003, 2005; Hakura et al., 2009; Abdih et al., 2012).

### Remittance and Openness

The increasing value of remittances and their beneficial effect on the receiving countries' economic conditions give their policymakers good incentives to promote these flows' attractiveness. Among the potential schemes to improve these revenues, the widening of financial boundaries is a possible strategy tool for governments. By lowering the cost of remittances sent through official means or relaxing the limitation of financial flows from abroad, policymakers may significantly increase the overall amount of funds collected. Financial transparency, though, poses new costs and challenges for the receiving countries.

A study was performed to assess the impact of remittance trade openness in Nepal by applying time-series data. Study findings reveal a positive association and confirm the critical role in increasing income level through domestic trade expansion. In addition, Gnangnon (2020) gauges the impact of remittances on foreign direct investment by considering a panel of 116 countries. Study findings reveal that trade openness and foreign capital inflows are positively induced by the recipients of remittance inflows in the economy.

### Remittances and Financial Stability

Remittance's role in the financial system has been extensively investigated through divers measures, such as financial inclusion (Aggarwal et al., 2006; Chowdhury, 2011; Anetor, 2019; Sobiech, 2019) and financial innovation. Despite the increasing value of remittances, little research has been done on the relationship between remittances and financial access. Unbanked remittance recipients, according to Inoue (2019), can demand secure storage of surplus money from banks and non-bank financial institutions, as well as other financial products and services. A study argued that remittances produce complementary effects in the financial sector by establishing operational efficiency and consistency through economic augmentation. Remittances prefer to work in reconstruction and restructuring in the financial sector; Mundaca (2009) advocated that the complementary role of remittances facilitates economic growth with financial efficiency. Giuliano and Ruiz-Arranz (2009) demonstrate that in countries where the financial sector is underdeveloped, remittances reduce credit restrictions and act as a replacement for financial production, boosting resource distribution and increasing economic growth.

On the other side, Mundaca (2009) specifies that financial developments could theoretically contribute to the greater use of remittances, thus encouraging progress. Aggarwal et al. (2011) indicate that remittances will directly foster non-financial growth. They found that remittances had a significant and optimistic effect on the GDP of bank deposits. Overall, the literature finds that the net effect on development seems optimistic, without considering the positive effects that remittances can have on income distribution. Ratha (2005) advocates remittance as a vital source of external capital inflows for achieving sustainability in financial progress. The study suggests that having a well-developed finance system and transport infrastructure will improve the transfer of money from one place to another and lead to increased structured transactions.

## Data and Methodology of the Study

The aim of this study was to assess the impact of remittance on economic openness and financial stability in least developed countries by considering a panel of 40 countries for the period spanning 1975–2018. The selection of countries and sample period rely on data availability. As a dependent variable of the empirical estimation, remittances measured by personal remittances received (% of GDP) were extracted from world development indicators. Regarding independent variables in the empirical assessment, the study considers economic oppressors and financial stability; see Table 1 for a summary of proxy formation of variables.

**Table 1:** Variables definitions and notation in the equation.

**Variables**	**Notation**	**Definition**
Remittance	RE	Personal remittances received (% of GDP)
Openness	FO	Financial openness is classified according to three regimes (closed, neutral, or open) based on the KAOPEN financial openness indicator of Chinn and Ito (2008).
	EO	Foreign direct investment, net inflows (% of GDP)
		Total trade (sum of imports and exports) as a % of GDP
Financial stability	FS	M2 to GDP
Government debt	D	External debt stocks, long-term (DOD, current US$)
Capital formation	GCF	Gross capital formation (% of GDP)
Financial development	FD	Domestic credit to the private sector (% of GDP)
Economic growth	Y	GDP per capita (constant 2010 US$)

### Openness (O)

The terms “economic globalization” and “openness” are often interchanged. In the related literature, though, transparency is the most popular concept for capturing the increasing foreign convergence of exchange and finance. We like to use it as the term “globalization” (Amna Intisar et al., 2020). Existing metrics of economic openness, commonly interpreted as the degree to which non-domestic players may or do engage in a domestic economy, may be grouped in two ways: first, according to the form of openness—“physical” or “financial”—they seek to quantify and, second, according to the sources used, to compose the measure of openness. These sources are either aggregated economic figures (de-facto measures) or evaluations of economic transparency's structural basis, i.e., lawfully defined obstacles to exchange and financial transactions. Addressing economic openness in literature, two lines of the study available are one group of researchers have considered total trade as a percentage of GDP as a measure of EO, see Fujii (2019) and Chen et al. (2021), and simultaneously another line of study has been considering FDI inflows as a measured of EO, see Steiner and Saadma (2016), Baltagi et al. (2009). Furthermore, financial openness is classified according to three regimes (closed, neutral, or open) based on the KAOPEN financial openness indicator of Chinn and Ito (2008).

### Financial Stability

In related literature, several proxies for financial stability have been employed, e.g., monetary aggregates such as M2 to GDP or financial intermediation indices such as the ratio of domestic credit to the private sector to GDP. However, there is no consensus on the superiority of any of these indicators to date, following the recent examples by Ang and McKibbin (2007) and Gries et al. (2009).

Apart from dependent and independent variables, the study considers government debt, gross capital formation, financial development, and economic growth as control variables. The motivation to include the variables mentioned above in the equation is to enrich the estimation efficiency and consistency. The selected variables have contributed to achieving the state of both economic and financial openness (Yasmeen et al., 2021), simultaneously triggering the growth of financial stability (Broner et al., 2011).

The generalized model based on research variables is as follows:

(1) $$\begin{array}{l} {X*}_{i,t}=\beta_{1}{X*}_{i,t-1}+\beta_{2}R_{i,t}+\beta_{3}FD_{i,t}+\beta_{4}GCF_{i,t}\\ +\beta_{5}D_{i,t}+\beta_{6}Y_{i,t}+\mu_{i,t} \end{array}$$

The above equation is dynamic due to incorporating the lagged value of the dependent variable in the equation. Here, X^*^ specifies the representation of proxy measures for openness, which is measured by the KAOPEN index, FDI inflows, trade openness, and financial stability measures by M2 to GDP. R denotes remittance inflows. In the group of control variables present in the equation, FD denotes financial development, GCF stands for gross capital formation, D means external debt, and Y denotes economic growth par capital. The subscripts of i indicate cross-section units and *t* for time. The descriptive statistics of research variables display in Table 2.

**Table 2:** Descriptive statistics.

	**EO1**	**EO2**	**FO**	**R**	**D**	**GCF**	**DCP**	**Y**
**Panel-A: Descriptive statistics**								
Mean	−0.032	3.897	−0.757	0.219	21.322	2.885	2.459	6.495
Median	0.267	3.897	−1.218	0.632	21.306	2.902	2.499	6.379
Maximum	4.637	5.74	2.333	3.447	24.45	4.331	4.6	8.048
Minimum	−8.927	−1.787	−1.92	−7.012	17.679	−1.228	−0.909	5.301
Std. dev.	1.833	0.59	1.126	1.872	1.235	0.48	0.741	0.511
Skewness	−0.992	−3.433	1.778	−1.164	−0.07	−1.084	−0.279	0.32
Kurtosis	4.829	35.748	5.234	4.554	2.639	11.814	3.869	2.715
Jarque-Bera	214.135	32888.9	518.205	230.35	4.389	2420.744	31.42	14.453
Observations	842	705	705	705	705	705	705	705
**Panel-B: Pairwise correlation**								
EO1								
EO2	0.364							
FO	0.237	0.207						
R	0.078	0.094	0.251					
D	0.117	−0.154	−0.017	0.13				
GCF	0.219	0.226	0.012	0.103	0.302			
DCP	−0.019	0.29	0.035	0.276	0.16	0.465		
Y	0.12	0.084	0.219	0.216	0.093	0.397	0.336	

Several econometrical tools were applied for gauging the impact of remittances on openness and financial stability in LDCs for the period spanning 1975–2018, such as a test of heterogeneity, cross-sectional dependency, a panel unit root test following Pesaran (2007), and a conventional and error correction-based panel co-integration test (Westerlund, 2008). This study prefers to apply Generalized Moments of Methods (GMM hereafter) familiarized by Arellano and Bond (1991) and System–GMM initiated by Blundell and Bond (1998). Several benefits induce the selection of the above-mentioned econometric methodology, such as System-GMM estimation, an effective tool for dynamic panel data estimation (Baltagi et al., 2009). Furthermore, instead of other econometric methods, the GMM technique provides more efficient and reliable predictions (Baltagi, 2008). Furthermore, this approach considers the predicted association between the error term and the country fixed effects exacerbated in complex penal data, which has less time and fewer cross-sections (Nickell, 1981). The GMM methodological approach can be used to solve the potential endogeneity and heterogeneity problem. According to Omri and Sassi-Tmar (2015), the GMM also tackles omitted predictor prejudice and heteroskedasticity and produces the estimate's reliability. Resolving the present state in dynamic panel data estimation, Arellano and Bond (1991) and Arellano and Bover (1995) offered a basic approach, which was generalized and further developed by Blundell and Bond (1998), commonly known as System-GMM estimation. It is worth mentioning here that two-step GMM estimation is more efficient than single-stage estimation. The instrument validation was confirmed through the Sargan test (Sargan, 1958) and the Hausman test (Hansen and Singleton, 1982).

The generalized specification of the System–GMM at a level and after first difference are as follows:

(2) $$\begin{array}{l} {X*}_{i,t}=\beta_{1}{X*}_{i,t-1}+\beta_{2}R_{i,t}+\beta_{3}FD_{i,t}\\ +\beta_{4}GCF_{i,t}+\beta_{5}D_{i,t}+\beta_{6}Y_{i,t}+\varphi_{it}+\mu_{i,t} \end{array}$$

The difference form is:

(3) $$\begin{array}{l} X{*}_{i,t}-X_{it-1}=\beta_{1}(X{*}_{i,t-1}-X_{it-1})+\beta_{2}(R_{i,t}-R_{it-1})\\ +\beta_{3}(FD_{i,t}-FD_{it-1})+\beta_{4}(GCF_{i,t}-GCF_{it-1})+\beta_{5}(D_{i,t}-D_{it-1})\\ +\beta_{6}(Y_{i,t}-Y_{it-1})+(\varphi_{it}-\varphi_{it-1})+(\mu_{i,t}-\mu_{i,t-1}) \end{array}$$

### System-GMM-Based Panel Granger Causality Test

For specifying directional causality between financial development, trade openness, cross-broader capital flows, and renewable energy consumption, the study followed the panel error correction model causality test discussed by Shabani and Shahnazi (2019) and Qamruzzaman and Jianguo (2020a) in their research work. A panel Granger causality test with System-GMM application was performed in two steps. In the first step, the long-run model estimation was performed with Dynamic-OLS (DOLS) to retrieve the residuals. Second, the residual obtained from DOLS estimation is used as an error correction term with the lagged first difference, which specifies long-run causality in the model. The equations for the short-run and long-run causality estimation are presented below:

(4) $$\begin{array}{l} {\Delta X*}_{it}=\beta_{1i}+\overset{m}{\underset{k=1}{\sum }}\beta_{11ik}{X*}_{it-k}+\overset{m}{\underset{k=1}{\sum }}\beta_{12ik}R_{it-k}\\ +\overset{m}{\underset{k=1}{\sum }}\beta_{13ik}D_{it-k}+\overset{m}{\underset{k=1}{\sum }}\beta_{14ik}GCF_{it-k}+\zeta_{1i}ECT_{it-1}+e_{1it} \end{array}$$

Where p represents the optimal lag length, which is determined by using Akaike's information criterion (AIC), we found that optimal lag for the estimation is 2, ECT stands for error correction term for assessing long-run causality, and *e*_*it*_ for the error term.

The underlying principle of using the System–GMM in determining a causality test with panel error correction is consistent and unbiased in estimation. On the other hand, OLS-based estimation is biased and creates an endogeneity problem in estimation (Soto, 2009; Combes et al., 2017). Therefore, other econometric techniques are required.

The Generalized Method of Moments (GMM) is a commonly used econometric methodology in panel data estimation with endogenous regressors. In the empirical literature, there are two types of GMM estimations. The first difference GMM estimation proposed by Arellano and Bond (1991) and the System-GMM estimation proposed by Arellano and Bover (1995) and further developed by Blundell and Bond (1998). The first difference GMM estimation suffers from weak instruments and small sample sizes when endogenous variables are close to a random walk (Blundell and Bond, 1998). The emergence of System-GMM estimation overcomes the first difference in GMM estimation (Arellano, 2003; Baum et al., 2007; Baltagi, 2008; Han et al., 2014). The System-GMM performs by estimating two system equations. First, the original levels equation with a suitable lagged first difference is used as an instrument and the first difference equation with a suitable lagged level is used as an instrument. Second, the application of System-GMM reduces the finite sample bias and increases consistency in estimation (Blundell and Bond, 1998). Therefore, we performed System-GMM estimation by using a prior developed Equation (4).

The short-run and long-run causality, after System-GMM estimation, will be identified by applying a standard Wald test. The null hypothesis of no causality will be rejected if the coefficients of β_11_ to β_44_= 0 and the coefficient of statistically significant ECT ascertain the existence of long-run causality in the equation.

## Estimation and Interpretation

### Cross-Sectional Dependency Test, Heterogeneity, Panel Unit Root Test, and Co-integration

Before performing the econometric empirical model estimation for gauging the impact of remittance on openness and financial stability in least developed counties for the period spanning 1976–2018, the study performs an elementary assessment through cross-sectional dependency (CSD), the heterogeneity test, and the panel unit root test with both the first and second generation and the co-integration test for long-run association. The results of cross-sectional dependency are exhibited in Table 3. The associated *p*-value of test statistics ascertains that research units share a familiar dynamic among them. In addition to the CSD test, the study intends to evaluate heterogeneity following the framework familiarized by Pesaran and Yamagata (2008). The estimation results are displayed in Table 3, columns 5-6 with two coefficients, i.e., Δ and adjΔ. Study findings establish the availability of heterogeneous properties in the selected dataset by rejecting the null hypothesis of homogeneity at a 1% level of significance.

**Table 3:** Results of cross-sectional dependency test and heterogeneity test.

	**Cross-sectional dependency test**				**Heterogeneity test**	
	**LM_***BP***_ Breusch and Pagan (1980)**	**LM_***PS***_ Pesaran (2004)**	**CD_***PS***_ Pesaran (2006)**	**LM_***adj***_ Pesaran and Yamagata (2008)**	**Δ**	**Adj.Δ**
	**[1]**	**[2]**	**[3]**	**[4]**	**[5]**	**[6]**
*EO1*	322.981^***^	25.44^***^	164.06^***^	35.201^***^	91.11	75.373
*EO2*	425.093^***^	15.16^***^	195.08^***^	23.212^***^	45.862	121.32
*EO3*	308.188^***^	24.12^***^	111.12^***^	15.02^***^	15.526	89.584
*FS*	182.813^***^	19.23^***^	150.55^***^	28.416^***^	42.221	71.551
*R*	241.649^***^	17.68^***^	242.59^***^	35.167^***^	34.901	87.925
*GCF*	245.592^***^	30.97^***^	138.36**	54.619^***^	70.358	131.289
*FD*	255.103^***^	34.54^***^	147.35^***^	50.268^***^	84.339	91.038
*D*	295.589^***^	34.01^***^	184.09^***^	39.269^***^	74.327	136.052
*Y*	210.65^***^	25.01^***^	111.31^***^	40.75^***^	23.857	89.285

*The superscript of*

*** *denotes the level of significance at a 1%, respectively*.

Following, the study moves to assess the order of the integration of the variable by performing panel unit root tests following Levin et al. (2002) known as an LLC test, Im et al. (2003)—the IPS test, and the Fisher-ADF initiated by Maddala and Wu (1999) which have the null hypothesis that all the panel data contain a unit root. Results of the panel unit root tests are displayed in Table 4. Study findings established that variables are stationary either at a level or after the first difference; neither variable is stationary after the second difference.

**Table 4:** Test of unit root rest-first generation.

	**LLC t**		**IPS W-stat**		**ADF—Fisher Chi-square**	
	**t**	**t and c**	**t**	**t and c**	**t**	**t and c**
***Panel–A: Al level***						
*EO1*	−0.409	−1.806	−3.565^***^	−1.895	32.435	56.165^***^
*EO2*	−2.791^**^	−3.811^***^	−0.89	−2.602^**^	51.427^**^	37.836
*EO3*	−0.096	−0.913	−2.832	−1.291	53.172^***^	59.753^***^
*FS*	−1.511	−2.695^**^	−2.836^***^	−2.883^***^	38.758	53.561^***^
*R*	−2.784	−1.801	−2.375	−0.759	33.74	60.099^***^
*GCF*	−0.289	−2.878^***^	−0.257	−1.042	46.592	53.632^***^
*FD*	−0.649	−0.056	−0.831	−1.127	43.803	30.359
*D*	−0.24	−3.448^***^	−0.403	−3.951^***^	55.062^***^	39.567
*Y*	−0.56	−3.577^***^	−3.223	−2.203	46.212	33.819
***Panel–B: After the first difference***						
*ΔEO1*	−8.915^***^	−16.574^***^	−20.125^***^	−7.858^***^	193.588^**^	113.242^***^
*ΔEO2*	−8.648^***^	−16.81^***^	−14.991^***^	−6.548^***^	141.347^***^	83.029^***^
*ΔEO3*	−7.285^***^	−15.386^***^	−12.18^***^	−6.961^***^	235.898^***^	183.493^***^
*ΔFS*	−6.111^***^	−16.154^***^	−20.145^***^	−7.214^***^	267.739^***^	113.225^***^
*ΔR*	−9.722^***^	−5.196^***^	−17.484^***^	−9.796^***^	132.651^***^	141.496^***^
*ΔGCF*	−9.998^***^	−12.052^***^	−18.634^***^	−6.815^***^	195.357^***^	116.565^***^
*ΔFD*	−10.367^***^	−12.035^***^	−18.853^***^	−5.586^***^	134.13^***^	199.964^***^
*ΔD*	−9.901^***^	−14.625^***^	−16.267^***^	−6.643^***^	227.717^***^	159.216^***^
*ΔY*	−10.767^***^	−19.792^***^	−8.249^***^	−8.593^***^	287.018^***^	187.628^***^

*The superscript of*

**, *** *denotes the level of significance at a 5% and 1%, respectively*.

Furthermore, considering the CSD test results, the study performs second generation panel unit root tests, commonly known as CIPS and CADA proposed by Pesaran (2007), which can efficiently handle the presence of typical dynamism. Results of the panel unit root tests are exhibited in Table 5. It is apparent from study findings that variables are integrated in a mixed order. However, neither variable is stationary after the second difference.

**Table 5:** Unit root test—second generation.

	**CIPS**				**CADF**			
	**At level**	**Δ**	**At level**	**Δ**	**At level**	**Δ**	**At level**	**Δ**
**EO1**	**C**	**CandT**	**C**	**CandT**	**C**	**CandT**	**C**	**CandT**
EO2	−1.485	−1.629^**^	−2.451^***^	−4.406^***^	−1.344	−2.005^***^	−2.381^***^	−6.347^***^
EO3	−1.633^**^	−1.11	−6.568^***^	−5.241^***^	−1.359	−2.518^***^	−4.034^***^	−4.181^***^
FS	−2.807^***^	−2.988	−4.769^***^	−7.207^***^	−2.199^***^	−2.717^***^	−2.645^***^	−4.467^***^
R	−1.287	−2.139	−7.643^***^	−4.894^***^	−2.676^***^	−1.371	−5.049^***^	−5.747^***^
GCF	−2.261	−2.815^***^	−6.768^***^	−5.917^***^	−2.271^***^	−1.972^**^	−4.652^***^	−5.516^***^
FD	−2.938^***^	−2.89^***^	−5.056^***^	−3.074^***^	−2.303^***^	−1.58	−6.907^***^	−2.571^***^
D	−1.307	−2.734^***^	−6.081^***^	−3.887^***^	−2.995^***^	−1.967^**^	−3.751^***^	−5.757^***^
Y	−2.956^***^	−2.953^***^	−2.028^***^	−2.686^***^	−1.153	−1.574	−3.126^***^	−5.75^***^

*The superscript of*

**, *** *denotes the level of significance at a 5% and 1%, respectively*.

In the next step, the long-run association between remittances, financial stability, and openness were investigated by performing a panel co-integration test offered by Pedroni (2001, 2004). Results of the panel co-integration test are displayed in Table 6, consisting of four empirical models based on proxy measures of openness and financial stability (see Table 1). The test statistics with the Pedroni panel co-integration test produce 11 outcomes. Most test outputs are statistically significant at a 1% level, indicating a long-run association in the equation. This conclusion applies to all four mode estimations. Furthermore, the ADF test statistics for gauging the long-run association were statistically significant at a 1% level, implying the rejection of the null hypothesis of no co-integration. Hence, it has convincingly established the availability of the long-run association between remittances, financial stability, and openness in least developed countries.

**Table 6:** Panel co-integration test.

	**Models**			
	**[1]**	**[2]**	**[3]**	**[4]**
***Panel–A: Pedroni residual co-integration test***				
Panel v-Statistic	1.368	1.459	2.708^***^	1.403
Panel rho-Statistic	−4.502^***^	−5.711^***^	−5.407^***^	−5.132^***^
Panel PP-Statistic	−8.258^***^	−9.403^***^	−10.946^***^	−10.187^***^
Panel ADF-Statistic	−4.529^***^	−0.695	−4.417^***^	−5.122^***^
Panel v-Statistic	−0.721	−1.262	−5.855^***^	−1.103
Panel rho-Statistic	−7.358^***^	−7.182^***^	−0.215^***^	−11.84^***^
Panel PP-Statistic	−6.986^***^	−10.305^***^	−1.161	−10.107^***^
Panel ADF-Statistic	−11.063^***^	−7.819^***^	−8.558^***^	−0.859
Group rho-Statistic	−11.989^***^	−6.193^***^	−8.025^***^	−7.127^***^
Group PP-Statistic	−7.508^***^	−11.099^***^	−6.023^***^	−0.914
Group ADF-Statistic	−3.846^***^	−3.677^***^	−2.599	−2.705
***Panel–B: Kao residual co-integration test***				
ADF	−2.9726^***^	−1.5814^***^	−2.8971^***^	−5.8228^***^

*The superscript of*

**, *** *denotes the level of significance at a 5% and 1%, respectively*.

Furthermore, the study performed the newly emerged panel co-integration test with the error correction term familiarized by Westerlund (2008). Results of the panel co-integration test are reported in Table 7 It is apparent from the test statistics that all the associated *p*-values are statistically significant at a 1% level. These findings suggest the existence of a long-run association between remittances, financial stability, and openness.

**Table 7:** Results of Westerlund (2008) panel co-integration test.

**Empirical model**	**Gt**	**Ga**	**Pt**	**Pa**
[1]	−11.373^***^	−13.71^***^	−15.911^***^	−13.507^***^
[2]	−4.524^***^	−8.826^***^	−11.623^***^	−9.02^***^
[3]	−7.872^***^	−8.02^***^	−12.848^***^	−6.014^***^
[4]	−9.831^***^	−14.15^***^	−10.371^***^	−7.206^***^

*The superscript of*

*** *denotes the level of significance at a 1%, respectively*.

The following results of empirical model estimation following GMM and System-GMM frameworks are displayed in Table 8. Coefficients for GMM estimation are reported in Panel-A and System-GMM coefficients are in Panel-B.

**Table 8:** Results of model estimation with GMM and System-GMM.

	**[1]**	**[2]**	**[3]**	**[4]**
***Panel-A: GMM estimation***				
R	0.128^***^[16.206]	0.248^*^[27.248]	0.137^***^[41.985]	0.043^***^[25.178]
GCF	0.034^**^[11.742]	0.053^**^[6.078]	0.098^**^[22.861]	0.191^***^[5.096]
D	−0.022^**^[−4.754]	−0.169^***^[−4.656]	−0.029^***^[−6.618]	0.053^***^[6.115]
FD	0.027^***^[2.061]	0.019^***^[4.985]	0.061^**^[1.892]	0.081^**^[3.301]
Y	0.066^***^[4.382]	0.122^***^[12.762]	0.033^***^[12.979]	0.099^***^[27.014]
***Panel-B: System-GMM estimation***				
EO(-1)	0.071^***^[105.826]			
FDII(-1)		0.094^***^[25.509]		
FIo(-1)			0.072^***^[14.524]	
FS(-1)				0.035^***^[76.872]
R	0.155^***^[1.262]	0.074^***^[2.874]	0.116^***^[10.283]	0.061^***^[12.031]
GCF	0.053^***^[31.026]	0.071^***^[13.117]	0.083^***^[12.794]	0.044^***^[23.325]
D	−0.091[−10.811]	−0.055^***^[−1.998]	−0.192^***^[−2.515]	0.041^***^[1.303]
FD	0.0174^***^[0.3077]	−0.192^***^[−2.563]	−0.188^**^[−1.179]	−0.016[−1.195]
Y	0.025^***^[10.628]	0.097^***^[21.127]	0.446^**^[11.735]	0.057^**^[7.483]
AR(1)	0.001	0.000	0.002	0.0001
AR(2)	0.745	0.557	0.784	0.754
Sargan test	0.541	0.984	1.000	0.884
Hansen test (*p*-value)	0.774	0.881	0.441	0.512

*The superscript of*

*, **, *** *denotes the level of significance at a 10%, 5%, and 1%, respectively*.

The coefficient of remittances exposes positive statistically significant impacts running toward the measures of openness, i.e., economic openness (a coefficient of 0.128 in column 1 and a coefficient of 0.248 in column 2) and financial openness (a coefficient of 0.137) and financial stability (a coefficient of 0.043 in column 4). Study findings suggest that continual inflows of remittances in the economy can cause economic openness and financial openness to thrive and establish financial stability in the financial system. Furthermore, the System-GMM estimation establishes the similar line of association between remittances, openness, and financial stability, that is, positive effects running from remittances to openness (see, panel –B, a coefficient of 0.155 in column 1; a coefficient of 0.074 in column 2; a coefficient of 0.116 in column 3) and financial stability (a coefficient of 0.061). Study findings establish that for boosting and augmenting openness in the economy through economic openness and financial openness and achieving financial stability in the financial system, remittance inflows play a critical role. Study findings are in line with Beine et al. (2012) and Bang et al. (2015). Only “more open” nations seemed to benefit from remittances (Dastidar, 2017), implying that remittances are insufficient for development on their own. The size of the benefit is determined by the recipient country's internal institutions and macro-economic situation. Unlike “less open” nations, “more open” nations have superior institutions and financial markets to take advantage of remittance revenue and channel it into successful investments, accelerating economic development.

The impact of domestic gross capital formation exposes a positive statistically significant association with economic openness (a coefficient of 0.034 in column 1 and a coefficient of 0.053 in column 2), financial openness (a coefficient of 0.098), and financial stability (a coefficient of 0.19) in GMM estimation. Moreover, a similar association line was established with System–GMM estimation, that is positive statistically significant impacts running from capital formation to economic and financial openness and financial stability. Study findings are supported by empirical literature; see Solarin and Shahbaz (2015), Yasmeen et al. (2021), Avelino et al. (2005). On the other hand, the elasticity of government debt disclosed a negative statistically significant association with openness and financial stability, available in empirical model estimation.

The directional association between remittance inflows, openness, and financial stability is investigated by performing the Granger causality test with System-GMM specification. The panel causality test results are displayed in Table 9 with four outputs based on different proxies of openness and financial stability in the equation.

**Table 9:** Granger causality test with System-GMM specification.

	**EO**	**R**	**FD**	**D**	**GCF**	**Y**	**ECT(-1)**
**Panel—A: Dependent variable- openness measured by KAOPEN index**							
EO	–	8.504^**^	13.542	6.321^*^	1.311	2.424	−0.531^***^
R	7.928^**^	–	2.192	0.205	0.644	0.734	−0.045
FD	0.76	1.62	–	5.999^*^	7.236^**^	5.896^**^	0.189
D	2.696	4.312^*^	3.037	–	5.064^*^	25.497^***^	0.929
GCF	0.675	7.465^**^	2.309	0.651	–	4.526^*^	0.272
Y	16.079^***^	7.454^**^	19.669^***^	3.319	11.879^***^	–	−0.541^***^
**Panel–B: Dependent variable-openness measured by FDI inflows**							
EO	–	11.376^***^	1.062	6.919^**^	0.242	1.493	−0.613^***^
R	6.896^**^	–	7.34^**^	0.033	3.04	5.164^**^	0.651
FS	0.791	3.792^*^	–	3.567^*^	3.727^*^	2.557	−0.245^***^
D	9.613^***^	10.181^***^	2.298	–	1.538	13.992^***^	0.155
GCF	1.756	2.232	1.183	0.311	–	4.634^*^	0.045
Y	7.293^**^	3.535^*^	13.594^***^	3.361	5.663^**^	–	0.142
**Panel–C: Dependent variable-openness measured by FDI outflows**							
FO	0	0.998	4.212^*^	18.355^***^	18.972^***^	0.603	−0.201^***^
R	12.874^***^	0	10.289^***^	0.434	6.774^**^	6.989^**^	−0.172^***^
FS	4.487^*^	2.044	0	3.683^*^	13.931^***^	1.07	0.421
D	10.169^***^	2.536	2.351	0	2.752	1.749	0.557
GCF	6.333^**^	6.841^**^	1.561	2.595	0	1.404	0.248
Y	4.037^*^	2.203	29.003^***^	0.689	9.303^***^	0	−0.445^***^
**Panel–D: Dependent variable-financial stability**							
FS	–	12.697^**^	11.591^***^	1.65	0.572	19.595^***^	−0.593^***^
R	27.83^***^	–	0.23	1.697	3.259^*^	16.647^***^	−0.066
FS	7.495^**^	4.463^*^	–	0.436	3.436^*^	5.542^**^	0.561
D	5.206^**^	1.423	4.75	–	0.408	0.658	0.284
GCF	4.055^*^	7.739^**^	1.091	0.268	–	2.671	−0.572^**^
Y	10.873^***^	9.037^***^	21.57^***^	0.894	2.512	–	−0.882^**^

*The superscript of*

*, **, *** *denotes the level of significance at a 10%, 5%, and 1%, respectively*.

The summary results of directional causality are displayed in Table 10. For column 1, economic openness is measured by the KAOPEN index and results disclose bidirectional causality running between remittance inflows and economic openness [EO←→R] and government debt and economic openness [EO←→D]. Furthermore, unidirectional causality running from financial development to economic openness [FD→EO] and economic openness to economic growth [EO→Y] are apparent. For column 2, economic openness is measured by trade openness. The study reveals unidirectional causality running from economic openness to remittances [EO→R], government debt to economic openness [D→EO], and economic openness to economic growth [OE→Y].

**Table 10:** Summary results of causality test.

**Causalities**	**[1]**	**[2]**	**[3]**	**[4]**
X **←**≠ **→** R	←→	→	←→	←→
X **←**≠ **→**DEBT	←→	←	←→	←→
X **←**≠ **→**GCF	←	←→	←→	→
X **←**≠ **→**FD	←	na	←→	←
X **←**≠ **→**Y	→	→	→	←→
R **←**≠ **→**FD	na	←→	←	→
R **←**≠ **→**DEBT	-→	→	na	na
R **←**≠ **→**GCF	→	na	←→	←→
R **←**≠ **→**Y	→	←→	←	←→
FD **←**≠ **→**DEBT	←	←	←	
FD **←**≠ **→**GCF	←	←	←	←
FD **←**≠ **→**Y	←→	→	→	←→
DEBT **←**≠ **→**GCF	←	na	na	na
DEBT **←**≠ **→**Y	←	←	na	na
GCF **←**≠ **→**Y	←→	←→	→	na

*←→ explains bidirectional causality, → denotes unidirectional causality, and na means no causality between them*.

The causal effects of financial openness, measured by inflows of FDI, are reported in column 3. The study divulges bidirectional causality running between remittances and financial openness [R←→FO], government debt and financial openness [D←→FO], financial development and financial openness [FD←→FO], and domestic capital formation and financial openness [GCF←→FO]. Furthermore, unidirectional causality running from financial openness to economic growth [FO → Y] is apparent. A directional causal association with financial stability as a deepened variable is displayed in column 4. Study findings reveal bidirectional causality running between remittance and financial stability [R←→FS], financial stability and government debt [F←D], and financial stability and economic growth [Y←→FS]. Moreover, the study unveils unidirectional causality running from financial development to financial stability [FD←FS] and financial stability to gross capital formation [FS → GCF].

In a nutshell, directional causality exposes remittance inflows in the economy that play a critical role in the thriving process of augmenting economic openness, financial openness, and financial stability. This suggests that efficient channelizing of remittances in the economy can result in broader aspects for macro development.

### Robustness Test

Next, the study performs an empirical model coefficients robustness test by implementing the dynamic fixed effects model introduced by Alcántara and Padilla (2009) in panel form. Study findings reveal that each co-efficients' sign and significance align with System-GMM estimation, which is the study's prime output (please see, Table 11).

**Table 11:** Results of dynamic fixed effects: Remittance effects on openness and financial stability.

R	0.028^***^	[14.751]	0.289531	[7.150]	−0.14838	[−1.57803]	−0.00935	[−1.481]
DCP	0.082^***^	[12.413]	−0.57102	[−4.997]	−0.42021	[−1.64707]	0.061169	[3.434]
DEBT	0.013**	[5.567]	0.068881	[0.760]	−0.07384	[−0.364]	0.00474	[0.339]
GCF	−0.221^***^	[−3.363]	0.752^***^	[4.954329]	0.49206	[1.666]	0.199474	[8.459]
Y	0.413^***^	[3.1931]	2.444^***^	[7.545026]	3.949192	[5.223]	0.261332	[5.219]
C	−6.956^***^	[−8.072]	−22.9405	[−11.2933]	−23.7527	[−5.742]	1.558031	[4.780]

X*(−1) denotes the lagged value of the dependent variable in the equation. The superscript of

*** *denotes the level of significance at a 1%, respectively*.

## Findings and Conclusion

The role of remittances in the economy has been extensively investigated by considering diverse facts from both micro and macro perspectives, but any conclusive relationships are yet to be exposed. The impact of remittances on the economy immensely relies upon the socio-economic status of the countries. The study evaluates remittances' role in promoting openness: economic openness and financial openness and financial stability in least developed countries for the period spanning 1975–2018. The study implements several econometrical tools to gauge the impact of remittances, such as panel unit root tests following Pesaran (2007), error correction-based panel contention test following Westerlund (2008), GMM and System-GMM for elasticity derivation, and causality with System-GMM specificity by following Arellano and Bover (1995) and Blundell and Bond (1998). The key findings of the study are stated below:

*First*, the cross-sectional dependency test results confirm the presence of common dynamics among the research variable units. Moreover, the heterogeneity test establishes the rejection of homogeneity among units of the study. Second, referring to panel unit root test results, it is apparent that variables are integrated in a mixed order, indicating that they are stationary either at a level, I (0), and/or after first difference I (1), but neither variable is stationary after second difference.

Second, the long-run association in the empirical model is investigated through a panel co-integration test following Kao (1999); Pedroni (2004), and Westerlund (2008). The associated *p*-value of test statistics rejects the null hypothesis of “no co-integration,” implying that the common co-movement and dynamic available in the equation is apparent in the long run.

Third, empirical model estimation with GMM and System-GMM exposes positive statistically significant effects running from remittance inflows to the measure of openness, i.e., economic openness and financial openness. These findings are in line with Beine et al. (2012). The greater the volume of remittances obtained by a government, the more likely it is to be financially open. The beneficial impact of remittance is statistically important and economically significant. Counterfactual tests prove that the risk of being financially open is higher in countries that obtain a significant amount of remittances. Foad (2010) postulates that remittance inflows result in a positive acceleration in domestic trade, thus allowing trade liberalization by boosting economic activities due to trade openness.

Moreover, the positive statistically significant association is established between remittance inflows and financial stability which is supported by empirical studies, see Ratha (2005, 2007); Ratha and Mohapatra (2007); De and Ratha (2012). Recipients often invest in remittances, particularly in countries with stable economic policies. Policy reform and the easing of foreign exchange restrictions could have facilitated the usage of remittances (Muneeb et al., 2021). Countries could improve remittance inflows by improving financial sector infrastructure and promoting foreign travel, putting more funds into structured networks. Domestic capital formation's impact reveals a statistically significant positive association with openness and financial stability, observed in econometric model estimation. Study findings suggest that capital adequacy in the economy acts as a motivational factor for boosting economic expansion in both economic openness and financial openness.

Furthermore, capital formation fosters the financial sector's growth, assisting in achieving stability in the financial sector in the long run. On the other hand, excessive growth relies on the government's external debt which adversely causes financial openness and financial stability. However, a statistically insignificant association is established with economic openness. Therefore, the study finding suggests that the government's external debt indicates domestic inefficiency for capital formation, and debt excessiveness has an immense disadvantageous consequence for the economy. Furthermore, external debt causes instability in the exchange rate and domestic consumption and eventually instability in the economy (Kumar, 2019).

Fourth, the results of directional causality establish a feedback hypothesis that explains the causality between remittance and openness [EO←→R; FO←→R] and remittance and financial stability [RF←→S]. Study findings suggest that remittance inflows are critical for accelerating economic and financial openness, indicating that migrants prefer to remit their earnings to the home economy with a perceived belief that investment opportunity is available with a stable financial sector.

On top of the above, remittances' role is significantly crucial for overall economic progress, including economic openness, financial openness, and financial stability, especially in least developing countries. To avail the best possible results from remittances in LDCs, it is imperative to concentrate on the management and efficient channelization of migrants' remittances into productive areas. This can be through financial reformation, efficient intermediation, institutional development, and, most importantly, government intention. It is suggested that remittances from migrants can be intensified by offering motivational incentives such as a reduction of exchange control policies, financial incentives for remittance recipients, and congenial investment ambiance in the financial sectors. In addition, countries might improve remittance flows and transfer more cash into formal channels by enhancing financial infrastructure and enabling international travel.

Furthermore, facilitating international labor mobility is an even more important—and contentious—method of boosting remittance inflows to poorer nations. Increased international migration might provide significant advantages to the global economy. On the other hand, developed nations are apprehensive of loosening immigration restrictions, fearing that it would boost competition in local employment markets and place a budgetary burden on local taxpayers. Developed nations are also concerned that mass immigration would corrode cultural norms and jeopardize national security.

The empirical study does not have an inherent limitation in methodological assessment or factor integration in the model. However, future studies can be performed with non-linear assessment, which is extensively considered an empirical estimation. Furthermore, the incorporation of the variable, namely economic policy uncertainty has been extensively considered in assessing the impact of macro fundamentals, see (Adams et al., 2020; Udi et al., 2020; Jia et al., 2021; Xu et al., 2021) thus it is suggested for a future study. It may create an avenue for exploring new insights by incorporating EPU in the empirical assessment.

## Data Availability Statement

The original contributions presented in the study are included in the article/Supplementary Material, further inquiries can be directed to the corresponding authors.

## Author Contributions

MM: Introduction, methodology, and first draft preparation. MQ: Introduction, methodology, empirical model estimation, and final preparation. All authors contributed to the article and approved the submitted version.

## Conflict of Interest

The authors declare that the research was conducted in the absence of any commercial or financial relationships that could be construed as a potential conflict of interest.

## Publisher's Note

All claims expressed in this article are solely those of the authors and do not necessarily represent those of their affiliated organizations, or those of the publisher, the editors and the reviewers. Any product that may be evaluated in this article, or claim that may be made by its manufacturer, is not guaranteed or endorsed by the publisher.
