# Supplementary material for: Dose Remittances Matter for Openness and Financial Stability: Evidence From Least Developed Economies
Source: Front Psychol. 2021 Aug 10;12:696600. doi: 10.3389/fpsyg.2021.696600 (PMC8382878; doi:10.3389/fpsyg.2021.696600)
Supplement: Supplementary file 2 [file Data_Sheet_1.docx]

Economic Openness

Remittances

Gross capital formation

Debt

🡨🡪: Bidirectional causality

🡪: Unidirectional causality

Fig 1: Graphical abstract: EO measures by KEOPEN index

Economic Openness

Remittances

Gross capital formation

Debt

🡨🡪: Bidirectional causality

🡪: Unidirectional causality

Fig 2: Graphical abstract: EO measures by Trade openness

Financial Openness

Remittances

Gross capital formation

Debt

🡨🡪: Bidirectional causality

🡪: Unidirectional causality

Fig 3: Graphical abstract: FO measures by inflows of FDI

Financial stability

Remittances

Gross capital formation

Debt

🡨🡪: Bidirectional causality

🡪: Unidirectional causality

Fig 4: Graphical abstract: Financial stability
